# Investigation of the Protective Effect of Probiotic *Lactobacillus plantarum* Ep-M17 on the Hepatopancreas of *Penaeus vannamei*

**DOI:** 10.1155/2024/8216782

**Published:** 2024-04-12

**Authors:** Xiaoman Hu, Wenlong Xu, Hao Li, Bowen Lu, Yang Du, Jiong Chen

**Affiliations:** ^1^State Key Laboratory for Managing Biotic and Chemical Threats to the Quality and Safety of Agro-products, School of Marine Sciences, Ningbo University, Ningbo 315211, China; ^2^Laboratory of Biochemistry and Molecular Biology, School of Marine Sciences, Ningbo University, Ningbo 315211, China; ^3^Key Laboratory of Applied Marine Biotechnology of Ministry of Education, Ningbo University, Ningbo 315211, China

## Abstract

Infection with the pathogenic bacterium *Vibrio parahaemolyticus* typically causes severe hepatopancreatic damage in *Penaeus vannamei*, often resulting in acute shrimp mortality. Therefore, protecting the shrimp's hepatopancreas is crucial for enhancing their disease resistance. Previous research has demonstrated that the probiotic strain *Lactobacillus plantarum* Ep-M17 inhibits the growth of *V. parahaemolyticus* E1 in vitro. However, it remains uncertain whether Ep-M17 can provide protective benefits to the shrimp's hepatopancreas. To address this knowledge gap, our present study investigated the histological changes, enzyme activity, gene transcription, and metabolite levels in the hepatopancreas of shrimp after a 4-week diet supplemented with Ep-M17. The results revealed that incorporating Ep-M17 into the shrimp's diet alleviated the damage by *V. parahaemolyticus* E1 infection in hepatopancreatic cells. In addition, the inclusion of Ep-M17 notably boosted the effectiveness of immunodigestive enzymes such as SOD, AKP, and CAT. Furthermore, Ep-M17 stimulated gene transcription in crucial immune response-related signalling pathways like the mitogen-activated protein kinase signaling pathway and the antigen processing and presentation pathway. Moreover, the incorporation of Ep-M17 into shrimp diets increased the levels of *β*-alanine, and histidine in the hepatopancreas, enhancing anti-inflammatory capacity and improving the shrimp's immune response. Overall, the results indicate that incorporating Ep-M17 into the diet can enhance shrimp disease resistance by bolstering both immune response and metabolic activity within the hepatopancreas. These results underscore the importance of probiotics in controlling aquatic animal diseases and highlight Ep-M17 as a promising dietary supplement for enhancing shrimp health and immunity in aquaculture.

## 1. Introduction

*Penaeus vannamei* is known for its delectable taste and high nutritional value, making it an excellent choice for high-density factory farming due to its tolerance to a wide range of salinities. This species of shrimp is currently the most widely cultivated globally, accounting for 83% of all farmed shrimp [1, 2]. However, the expansion and intensification of aquaculture practices have rendered *P. vannamei* highly susceptible to various diseases. In addition to the white spot syndrome virus (WSSV), a plethora of emerging bacterial, fungal, and parasitic diseases have inflicted significant economic losses on the shrimp industry [3]. One such emerging disease is acute hepatopancreas necrosis disease (AHPND), which has been spreading rapidly across global *P. vannamei* farming regions since 2009. This disease exhibits wide and swift dissemination, with mortality rates surpassing 90% and discharge rates reaching 80%, consequently leading to a decline in global shrimp production [4]. AHPND shrimp displays distinctive histopathological features, including extensive necrotic and detached hepatopancreatic duct epithelia cells in the absence of bacterial colonization. Current studies have identified a link between acute hepatopancreas necrosis and *V. parahaemolyticus* infection carrying virulent plasmids pVHvo [5, 6].

While the use of antibiotics and other chemical drugs is effective in disease control within aquaculture, it raises concerns about safety and environmental impact, thereby hindering the industry's sustainable development [7–10]. As a result, there is a pressing necessity to investigate alternative approaches that ensure safety while also being environmental friendly. Probiotics have emerged as a widely recognized substitute that delivers health benefits to the host when consumed in adequate quantities [11–13]. In aquaculture, abundant research has demonstrated the positive effects of probiotics on the growth performance and immune defenses of shrimp [14–16]. Lactic acid bacteria (LAB), the predominant member of the LAB family, are frequently employed as probiotics in aquaculture due to their anaerobic, nonspore-forming, and acid-tolerant properties [17]. Notably, lactic acid bacteria exhibit adaptability to a broad range of temperatures, from 2°C to 53°C, facilitating colonization in various animal hosts [18]. Research has indicated that lactic acid bacteria hold promise in enhancing the health and immune function of aquatic animals, attributed to their ability to produce antimicrobial compounds that inhibit the proliferation of harmful bacteria in both the animal intestines and the surrounding aquatic environment [19–22].

The hepatopancreas of shrimp plays a vital role in nutrient absorption, as well as carbon and nitrogen metabolism, making it susceptible to damage from pathogenic bacteria [23, 24]. In previous investigations, the addition of *L. plantarum* Ep-M17 to the diets of *P. vannamei* resulted in improvements in the shrimp's gut microbiota and increased their resilience against *V. parahaemolyticus* E1 infection. This intervention resulted in a Relative percent survival (RPS) of 76.9% [25]. However, it remains unknown whether Ep-M17 can alleviate hepatopancreatic damage caused by *V. parahaemolyticus*. Hence, the intention of this study was to explore the hepatopancreatic protective properties of *L. plantarum* Ep-M17 in shrimp. This was pursued through histological examination, enzyme activity assessments, as well as transcriptomic and metabolomic sequencing analyses. We hope that these findings will provide valuable insights for the development of probiotic additives for disease control in shrimp aquaculture and environmental purification.

## 2. Materials and Methods

### 2.1. Bacterial Cultures

The *L. plantarum* strain Ep-M17 (BioProjects: PRJNA871793) was obtained from the intestine of a pearl gentian grouper. The strain was preserved at −80°C according to Du et al. [25]. Initially, the preserved *L. plantarum* Ep-M17 strains were thawed at room temperature, and an appropriate amount of bacterial solution was pipetted into 10 mL of de Man, Rogosa, and Sharpe (MRS) liquid medium for overnight incubation at 37°C. Following, the culture was transferred to a larger volume of MRS liquid medium to expand the culture.

### 2.2. Shrimp and Trial Diet

The experimental shrimp, with an average length of 4.5–5.5 cm per individual, were acquired from Xiangshan Aquatic Development Co., Ltd. in Ningbo, Zhejiang, China. Preceding the experiment, the shrimp were given a 1-week acclimate period in the new environment during which they were fed a basal diet. There were a total of 600 healthy shrimp randomly dispersed among six aquariums, each with a volume of 400 L and containing 100 shrimp. The control group (CDG group) was fed a commercial basal diet comprising fishmeal, soybean meal, canola meal, peanut meal, and corn protein powder. For the experimental group research design group (RDG group), the preserved *L. plantarum* Ep-M17 strain was thawed at room temperature. Subsequently, 100 *μ*L of the strain was added to an MRS liquid medium for overnight incubation at 37°C. The culture was then transferred to a larger volume of MRS liquid medium for expansion. The cultured Ep-M17 strain was centrifuged, washed with PBS, and evenly sprayed onto the basal diet, making the final concentration of the bacteria reach 5 × 10^8^ CFU/g feed, and then fed to shrimp in the experimental RDG group. Both groups of shrimp were fed three times a day (8:00, 14:00, and 20:00) for a total of 4 weeks. During the feeding experiments, bait was provided at approximately 6% of the shrimp's body weight [26].

### 2.3. Challenge Assay

After four weeks of feeding the shrimp with Ep-M17, the challenge assay was conducted. During the test, the shrimp continued to consume the experimental diet. The *V. parahaemolyticus* E1 was obtained from a −80°C refrigerator, revived, and then inoculated at a concentration of 0.1% into fresh 2216E broth. The broth was then incubated at 28°C with continuous shaking for 18 hr. The resulting bacterial solution was centrifugated at 3,000 *g* for 10 min at 4°C. Based on preliminary experiments, it was determined that a concentration of 1.0 × 10^7^ CFU/mL of *V. parahaemolyticus* E1 was optimal. Therefore, the collected bacteria were diluted with sterile seawater to achieve a concentration of approximately 1.0 × 10^7^ CFU/mL. The infection of shrimp by immersion lasted for 48 hr, after which the hepatopancreas tissues from the surviving shrimp were taken for histopathology, enzyme activity, gene transcriptome, and metabolomics analyses.

### 2.4. Histopathology

Initially, three shrimp were randomly chosen from each group at three specific time intervals: 2 weeks and 4 weeks postfeeding with Ep-M17, followed by a 48-hr exposure to *V. parahaemolyticus* E1. Their hepatopancreases were carefully removed using a sterile scalpel. Subsequently, the samples underwent fixation in a 4% paraformaldehyde solution for a duration of 24 hr. Following fixation, the samples underwent dehydration using a series of graded alcohol concentrations (70%, 80%, 95%, and 100%) and were incorporated into paraffin. Subsequently, the tissue was sliced into 0.5 mm thick sections using a rotary sectioning machine and stained with hematoxylin-eosin. Finally, the samples were examined under a light microscope at a magnification of 200x to capture images.

### 2.5. Immunoenzymatic Activity Analysis

The hepatopancreas samples were dried using filter paper, weighed, and subsequently homogenized in a buffer solution according to the protocol provided by the enzyme-linked immunosorbent assay (ELISA) Assay Kit. Following homogenization, the samples were centrifuged at 3,000 *g* for 10 min, and the supernatant obtained after centrifugation was collected. The activities of alpha-amylase (AMS), digestive enzyme trypsin (TRY) and lipase (LIP), as well as alkaline phosphatase (AKP), catalase (CAT), and antioxidant enzyme superoxide dismutase (SOD) were measured according to the instructions provided in the kit. Subsequently, the absorbance at 450 nm was assessed for each well within a 96-well plate utilizing a Rayto RT-6100 ELISA plate reader.

### 2.6. RNA Separation and cDNA Library Construction

Following 4 weeks of cultivation, nine shrimp were randomly chosen from each group, with three shrimp selected from each aquarium for sampling purposes. Hepatopancreas tissues were excised using sterile scissors and forceps, washed with sterile PBS to remove impurities, and stored in liquid nitrogen for subsequent ribonucleic acid (RNA) extraction. Hepatopancreas samples were processed for total RNA extraction utilizing TRIzol reagent and the RNeasy Mini Kit. Subsequently, the concentration and purity of the extracted RNA were evaluated using a Nano 6000 detector from Bioanalytical systems. To synthesize cDNA, 30 *μ*g of high-quality RNA was used in each group.

To isolate mRNA, Oligo (dT) magnetic beads were employed for enrichment, while ion interruption was utilized to fragment the RNA into fragments of approximately 300 bp in length. The initial phase involved the use of reverse transcriptase and six-base random primers to synthesize the first strand of cDNA. Following that, the second cDNA strand was produced, using the initial cDNA strand as a template. PCR amplification was performed to increase the abundance of library fragments, and subsequent selection of the library was guided by fragment size. The libraries were then quality assessed using the Agilent 2100 Bioanalyzer, followed by the determination of the overall library concentration and potent library concentration. Libraries featuring unique index sequences were mixed in suitable proportions determined by their concentrations and the desired sequencing depth. These combined libraries were uniformly dispersed to a concentration of 2 nm and subsequently converted into single-stranded libraries via alkali denaturation. Following RNA extraction and purification, library preparation ensued. The prepared libraries were then subjected to sequencing using Next-Generation Sequencing (NGS) technology on the Illumina HiSeq platform, employing Paired-end (PE) sequencing methodology.

### 2.7. Illumina Sequencing and Bioinformatics Analysis

To obtain the most authentic sequencing data file, the raw image data from Illumina HiSeq 4000 sequencing are processed through Base Calling and converted to FASTQ format. The Q30 value corresponds to an error rate of 0.001. Using statistical methods, the quality fluctuations of reads under all sequencing cycles were counted to visualize the quality of sequencing experimental data from a macroscopic perspective. The quality of bases, which is influenced by many elements including the sequencer itself, sequencing reagents, and samples, is connected to the error rate of sequencing for RNA-seq technology. To determine if sequencing is of good or terrible quality, the algorithm can be used to obtain a comprehensive number called quality (Q).

The transcript sequences were obtained by scratch splicing the obtained high-quality sequences, clustering the transcripts, selecting the longest transcripts as Unigene, and finally using Unigene for subsequent GO (Gene Ontology), KEGG (Kyoto Encyclopedia of Genes and Genomes) and other databases for prediction. Simultaneously, the filtered sequences were set against the Unigene database to determine the Reads Count for each Unigene. Using the filtered sequences, alignment to the Unigene database was performed to derive the Reads Count corresponding to each Unigene. Subsequently, expression differences and enrichment analyses were carried out based on this data for the samples.

### 2.8. Chromatography-Mass Spectrometry Analysis

The thawing process for hepatopancreas samples was conducted gradually at 4°C. Following thawing, an adequate amount of sample was combined with a precooled solution consisting of methanol, acetonitrile, and water in a ratio of 2 : 2 : 1 (v/v). Following agitation, the mixture was subjected to sonication at a low temperature for 30 min, followed by a 10-min incubation at −20°C. Subsequently, centrifugation was performed at 14,000 *g* for 20 min at 4°C, resulting in the separation of the supernatant. The supernatant was then subjected to vacuum drying before being mixed with a 100 *μ*L solution of aqueous acetonitrile (with a ratio of acetonitrile to water of 1 : 1, v/v) for subsequent mass spectrometry analysis. After vortexing and centrifugation at 14,000 *g* for 15 min at 4°C, the resulting supernatant was utilized for further analysis.

#### 2.8.1. Chromatographic Analysis Conditions

The separation of samples was conducted using an Agilent 1290 Infinity LC (UHPLC) system furnished with a hydrophilic interaction liquid chromatography (HILIC) column, operating at a temperature of 25°C and a flow rate of 0.5 mL/min. Injection of samples was carried out with a capacity of 2 *μ*L. The mobile phase comprised water containing 25 mM ammonium acetate and 25 mM ammonia (referred to as mobile phase A), as well as acetonitrile (referred to as mobile phase B). The elution was performed using a gradient method: B was maintained at 95% from 0 to 0.5 min, followed by a linear change in B from 0.5 to 7 min (from 95% to 65%), and then a linear change in B from 7 to 8 min (from 65% to 40%). B was kept at 40% from 8 to 9 min, followed by a linear change in B from 9 to 9.1 min (from 40% to 95%). During the analysis, the proportion of mobile phase B was held constant at 95% between 9.1 and 12 min. Throughout the analysis, the samples were maintained at 4°C within the autosampler.

#### 2.8.2. Mass Spectrometry Conditions

The AB Triple TOF 6600 mass spectrometer was utilized to acquire both the primary and secondary spectra of the samples. Prior to mass spectrometry analysis, the UHPLC system (the Agilent 1290 Infinity LC) was used to separate the samples. Analysis of the samples was conducted utilizing both negative and positive ion modes of electrospray ionization (ESI). The instrument was arranged with the following parameters: Gas 1 and Gas 2 were both set to 60, the gas curtain (CUR) was kept at 30 psi, the ion source temperature was set to 600°C, and the spray voltage (ISVF) was adjusted to ±5,500 V for both negative and positive modes. The mass-to-charge ratio detection range was set to 60–1,000 Da for primary ion detection and 25–1,000 Da for secondary ion detection. During the analysis, the primary mass spectrometry scan had an accumulation time of 0.20 s/spectra, while for the secondary mass spectrometry, it was 0.05 s/spectra. Secondary mass spectra were obtained using data-dependent acquisition mode (IDA) and peak intensity value screening mode. The declustering voltage (DP) was set at ±60 V for both negative and positive modes, with collision energy maintained at 35 ± 15 eV. The dynamic exclusion of isotope ions range was set to 4 Da, and 10 fragment profiles were acquired per scan.

### 2.9. Metabolomic Data Analysis and Processing

The data obtained from the mass spectrometer were initially processed into .mzXML format using ProteoWizard software. Following this, peak alignment, retention time correction, and extraction of peak areas were performed using the XCMS software. The resulting data underwent metabolite structure identification, quality evaluation, and preprocessing. A range of analytical methods were applied, encompassing univariate statistical analysis, multidimensional statistical analysis, differential metabolite screening, differential metabolite correlation analysis, and KEGG pathway analysis. These approaches were utilized to detect and assess noteworthy disparities in metabolites. Following initial processing, the data underwent comprehensive analysis employing various techniques, such as univariate and multidimensional statistical analysis, differential metabolite correlation analysis, differential metabolite screening, and KEGG pathway analysis. These methods were utilized to identify and assess significant variations in the metabolites.

### 2.10. Statistical Analysis

The data were represented as mean ± standard deviation, and statistical analysis was conducted using SPSS 25.0 software. Independent samples *t*-tests were employed to assess mean differences across various experimental groups. Statistical significance was determined with a threshold of *P* < 0.05 indicating significance, and *P* < 0.01 denoting extreme statistical significance.

## 3. Results

### 3.1. Histology of the Hepatopancreas

**Figure 1:**
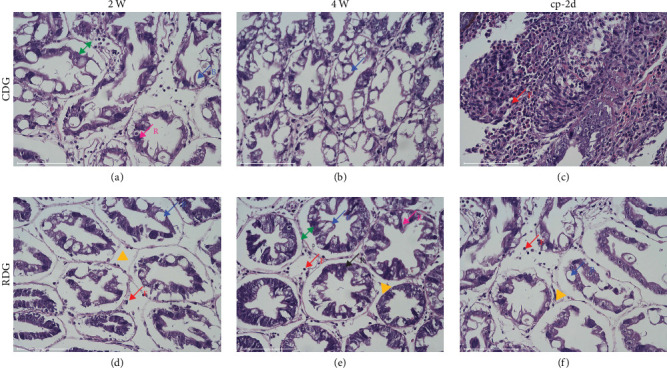
Hepatopancreas histology staining was performed using hematoxylin-eosin. Shrimp were divided into two groups and fed different diets: the CDG group fed basal diet, while the RDG group received a basal diet containing *L. plantarum* Ep-M17. Samples were collected at three different time points: the second (2 W) and fourth weeks (4 W) of feeding, and the 48 hr after the *V. parahaemolyticus* E1 attack (cp-2d). In the stained samples, R cells were observed to contain lipid vacuoles of varying sizes. F cells exhibited abundant ribosomes and well-developed endoplasmic reticulum. B cells typicall displayed a single large secretory vesicle. The green double arrows indicate the surface cell epithelium, while yellow triangles mark the cell interstices. Bar: 100 *μ*m. (a) Feeding with basal diet for the second week (2 W); (b) feeding with basal diet for the fourth week (4 W); (c) control group at 48 hours after *V. parahaemolyticus* E1 challenge (cp-2d); (d) feeding with diet supplemented with *L. plantarum* Ep-M17 for the second week (2 W); (e) feeding with diet supplemented with *L. plantarum* Ep-M17 for the fourth week (4 W); and (f) experimental group at 48 hours after *V. parahaemolyticus* E1 challenge (cp-2d).

Histological studies were conducted to examine the influence of Ep-M17 on the hepatopancreas structure of shrimp, both at the end of the feeding trial and the challenge assay. The results indicated that feeding Ep-M17 for 2 or 4 weeks led to a more intact hepatopancreatic basement membrane and a more regular stellate duct lumen structure in comparison to the control group (Figures 1(a), 1(b), 1(d) and 1(e)). In addition, following the *V. parahaemolyticus* E1 attack, the control shrimp exhibited severe cellular damage, including disruption of the luminal structure of the stellate ducts and a reduction in R and B cells (Figure 1(c)). However, the Ep-M17-fed shrimp maintained an intact hepatopancreatic basement membrane and the hepatic tubules were well preserved without any signs of cell necrosis or severe cellular damage (Figure 1(f)).

### 3.2. Immune and Digestive Enzyme Activity

**Figure 2:**
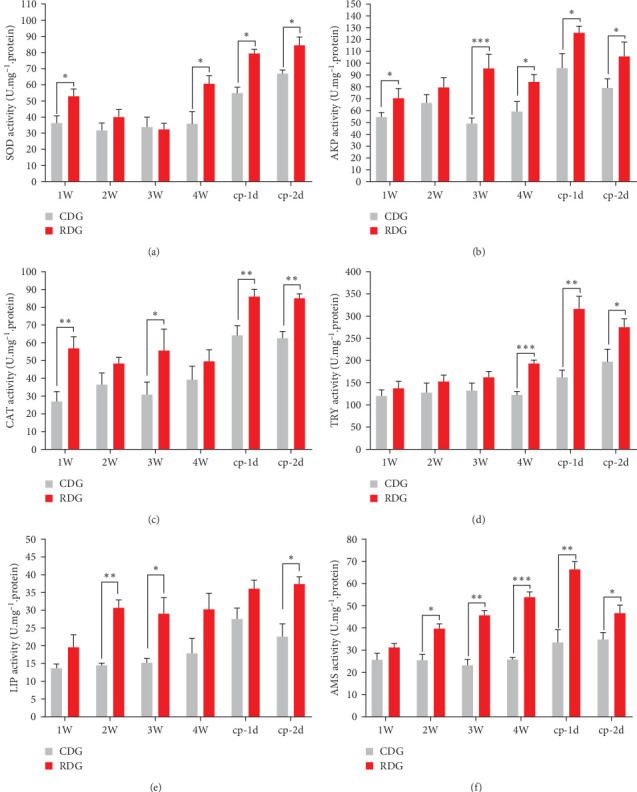
Measuring immune and digestive enzyme activity in *P. vannamei*'s hepatopancreas. (a) SOD; (b) AKP; (c) CAT; (d) TRY; (e) LIP; (f) AMS. The data are presented as mean values and standard deviation (±SD) for each parameter (*n* = 5). Different letters indicate significant differences between means in the same row (*⁣*^*∗*^*P* < 0.05, *⁣*^*∗∗*^*P* < 0.01, *⁣*^*∗∗∗*^*P* < 0.001). The *X*-axis represents the time points at 1, 2, 3, and 4 weeks after the start of the experiment, and cp-1d and cp-2d represent the 24 and 48 hr after the challenge, respectively.

The findings illustrated in Figure 2 suggest that the application of Ep-M17 probiotics to shrimp led to an elevation in the operation of immune- and digestion-related enzymes in the hepatopancreas. Within just one week of feeding, significant increases in the activity of SOD, AKP, and CAT were observed in the RDG group compared to the CDG group. Following the challenge with *V. parahaemolyticus* E1, the activity of AKP initially increased and then decreased in both groups, while the activity of SOD and CAT continued to increase. Notably, the activity of SOD, AKP, and CAT in the RDG group remained markedly greater than in the CDG group (Figure 2(a)–2(c)). In addition, after 2 weeks of feeding, the enzyme activities of LIP and AMS were significantly higher in the RDG group compared to the CDG group. TRY activity was also notably elevated in the RDG group after 4 weeks of feeding. Following the challenge with *V. parahaemolyticus* E1, TRY and AMS activities displayed a trend of rising and falling, whereas LIP activity continued to rise. Importantly, the enzyme activities of TRY, AMS, and LIP in the RDG group remained markedly greater than the CDG group (Figure 2 (d)–2(f)).

### 3.3. Transcriptome Sequence Assembly

Transcriptome sequencing and assembly were conducted on six cDNA libraries generated from mRNA isolated from shrimp hepatopancreas. The shrimp were fed either a control diet or a diet enriched with Ep-M17 probiotics. After eliminating low-quality reads and adapter sequences, the average number of clean reads obtained for the CDG and RDG groups was 43.53 million and 41.92 million, respectively. This yielded 41.22 million and 39.69 million high-quality reads, representing 94.66% and 94.78% of the original data, respectively (Table 1). The sequencing exhibited high quality, ensuring the suitability of the data for robust downstream analysis.

### 3.4. Identification of DEGs

**Figure 3:**
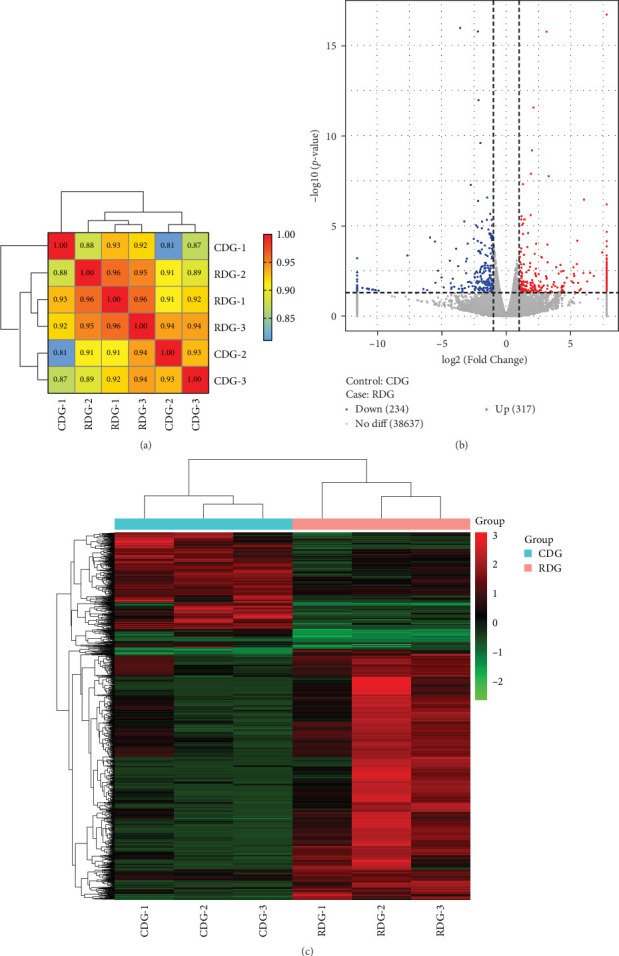
(a) Pearson correlation coefficients were calculated to assess the relationship among samples. (b) Volcano plot showing the differentially expressed genes in shrimp fed a basic commercial diet (CDG) compared to those supplemented with *L. plantarum* Ep-M17 (RDG). The *X*-axis represents the fold change in gene expression, while the *Y*-axis represents statistical significance. Red splashes indicate genes that are significantly upregulated, blue splashes indicate genes significantly downregulated, and shaded area splashes indicate genes without significant differences in expression. The corrected *P*-value is represented by—log_10_ (*P*-adjusted). (c) Heat map analysis.

Pearson's correlation coefficient was employed to assess the similarity in gene expression levels across the samples. The results showed that each sample exhibited a coefficient value >0.8, indicating a high degree of consistency in the gene expression patterns observed among the samples (Figure 3(a)). Differential expression genes (DEGs) were identified using DESeq, with a *P*-value threshold of <0.05 and |log2 fold change| > 1. The volcano plot in Figure 3(b) illustrates the correlation between the fold-change and *P*-value of DEGs that are either up or downregulated in each comparison. Compared to the CDG group, 38,637 unigenes exhibited no statistically significant variances, whereas shrimp fed with Ep-M17 demonstrated notable upregulation in 317 unigenes and downregulation in 234 unigenes. Cluster analysis of the transcriptional expression patterns revealed distinct clustering of CDG and RDG groups in the generated heat map (Figure 3(c)), indicating a significant influence of Ep-M17 supplementation on gene expression patterns in shrimp hepatopancreas.

### 3.5. GO Enrichment Analysis

**Figure 4:**
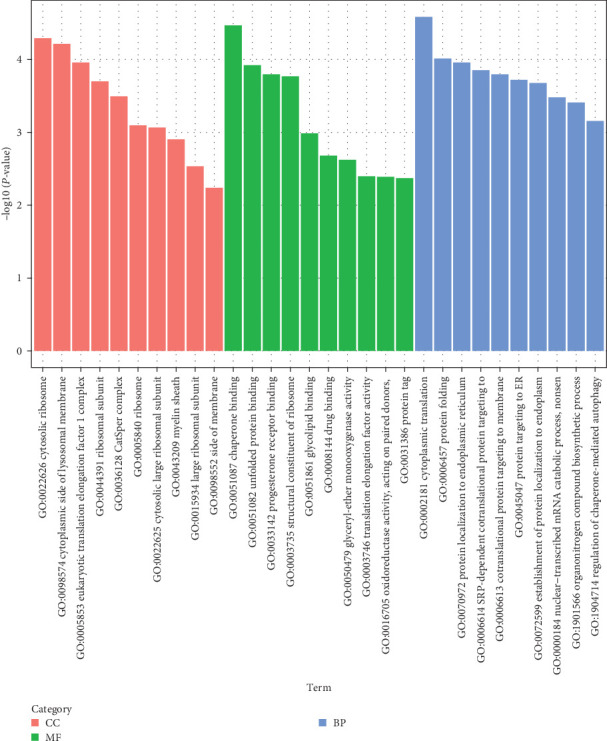
GO enrichment analysis of differentially expressed genes. Cellular component (CC), molecular function (MF), and biological process (BP).

Functional enrichment analysis using GO was conducted to determine the biological roles of the DEGs identified in the study (Figure 4). The analysis demonstrated that the upregulated DEGs after feeding Ep-M17 were predominantly enriched in the following categories, compared to the control group: cellular component (cytosolic ribosome, eukaryotic translation elongation factor 1 complex, cytoplasmic side of lysosomal membrane, ribosomal subunit, CatSper complex, ribosome, cytosolic large ribosomal subunit, myelin sheath, large ribosomal subunit, and side of membrane), molecular function (chaperone binding, unfolded protein binding, structural constituent of ribosome, progesterone receptor binding, glycolipid binding, drug binding, translation elongation factor activity, glyceryl-ether monooxygenase activity, acting on paired donors, oxidoreductase activity, and protein tag), and biological progress (cytoplasmic translation, protein folding, protein localization to endoplasmic reticulum, SRP-dependent cotranslational protein targeting, establishment of protein localization to endoplasm, protein targeting to ER, nuclear-transcribed mRNA catabolic process, cotranslational protein targeting to membrane, nonsen, organonitrogen compound biosynthetic process, and regulation of chaperone-mediated autophagy). According to the GO analysis, the immune genes that were found to be significantly differentially expressed in the hepatopancreas of shrimp fed Ep-M17 were mainly related to immune response, heat shock proteins, mitogen-activated protein kinase (MAPK) signaling pathway, and oxidoreductase activity (Table 2).

### 3.6. KEGG Enrichment Analysis

**Figure 5:**
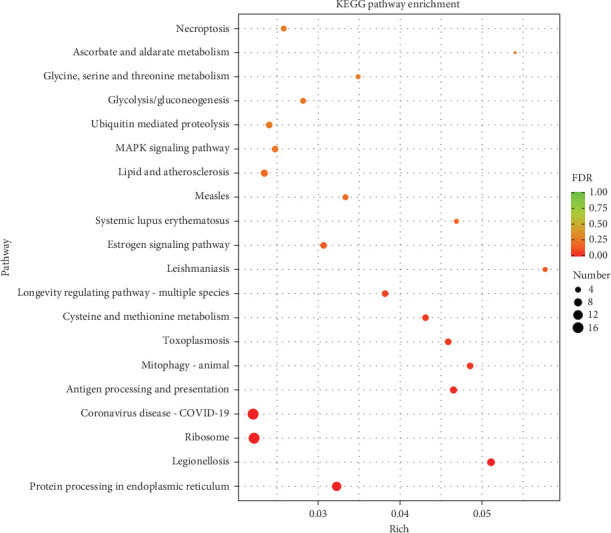
KEGG pathway analysis of the DEGs shown in scatterplots. The pathways are represented on the *Y*-axis, while the corresponding enrichment factor is represented on the *X*-axis. A darker shade of red indicates a smaller *P*-value, reflecting the significance of enrichment in a particular pathway. The size of the point indicates the number of DEGs in each pathway.

The top 20 KEGG pathways with the most significant enrichment, as determined by FDR values, were selected for display based on the results of KEGG enrichment analysis of differentially expressed genes. As depicted in Figure 5 (a) majority of the DEGs in the hepatopancreas of shrimp fed Ep-M17 showed enrichment in several pathways, including Protein processing in endoplasmic reticulum, Ribosome, Legionellosis, Antigen processing and presentation, Coronavirus disease-COVID-19, Mitophagy-animal, Toxoplasmosis, Cysteine and methionine metabolism, Longevity regulating pathway-multiple species, Leishmaniasis, Estrogen signaling pathway, Systemic lupus erythematosus, Measles, Lipid and atherosclerosis, MAPK signaling pathway, Ubiquitin mediated proteolysis, Glycolysis/Gluconeogenesis, Glycine, Ascorbate and aldarate metabolism, Necroptosis, serine and threonine metabolism, as compared to the control group. Further analysis displayed that immune-related genes exhibited significantly upregulated expression in each pathway (Table 3).

### 3.7. Metabolite Chemical Classification Attribution Statistics

**Figure 6:**
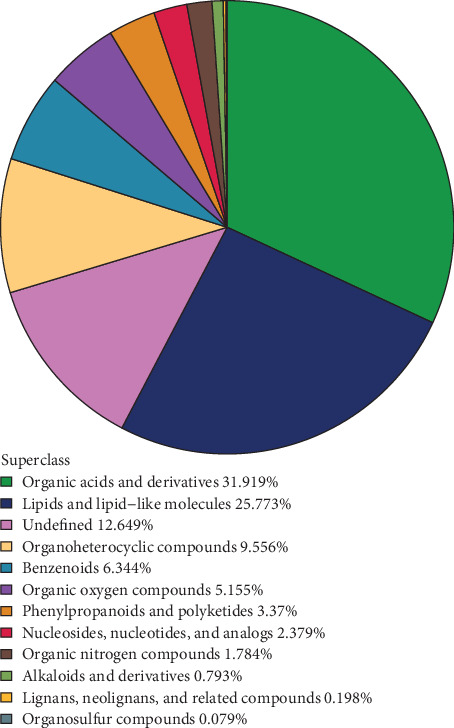
Metabolite chemical classification statistics. Each colored block represents a different chemical classification of metabolites, and the corresponding percentage indicates the proportion of metabolites in each category. The number of metabolites in each category is shown as a percentage of all identified metabolites. Metabolites that cannot be classified into any chemical category are labeled as “undefined”.

Chemical classification attribute information was employed to categorize all identified metabolites, encompassing both negative and positive ions. As illustrated in Figure 6, it was observed that the predominant components of the various metabolites in the hepatopancreas of shrimp fed Ep-M17 included organic acids and their derivatives, lipids and lipid-like molecules, along with other substances.

### 3.8. Analysis of Differences between Metabolic Groups

**Figure 7:**
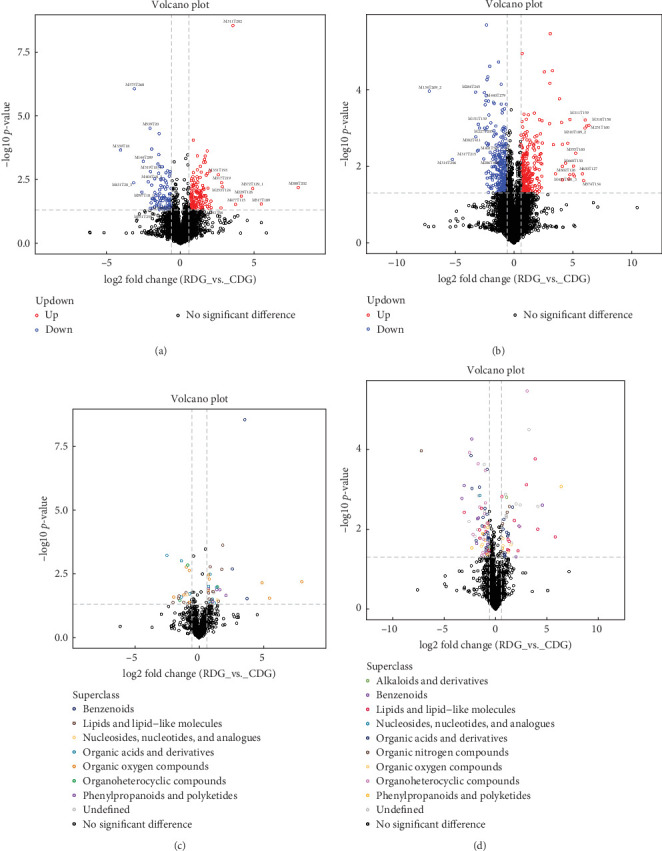
The differences between metabolic groups were analyzed using a negative/positive ion mode volcano map. (a and b): negative/positive ion mode volcano map. This plot shows the relationship between the fold change and the statistical significance of differentially expressed metabolites. The *X*-axis shows the fold change values and the *Y*-axis shows the—log_10_ transformed *P*-values. Metabolites that meet the criteria of FC > 1.5 and *P*-value < 0.05 are highlighted in red, while metabolites with FC < 0.05 are highlighted in blue. Metabolites that do not meet these criteria are shown in black. (c and d) negative/positive ion pattern volcano plot (colours correlate with differential metabolite chemical classification). The plot shows the logarithmic values of the fold change and the significance *P*-value for different metabolites. Metabolites with a fold change greater than 1.5 and a *P*-value less than 0.05 are shown in red, while metabolites with a fold change less than 0.05 are shown in blue. Metabolites that do not show significant differences are displayed in black. The chemical classification of metabolites is also represented by different colors.

**Figure 8:**
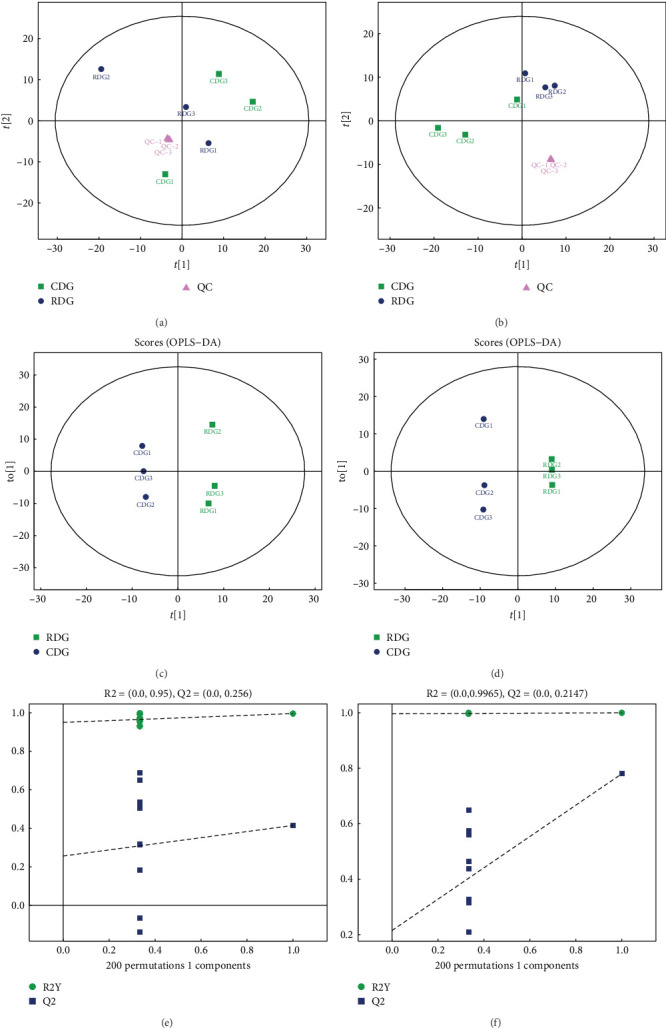
Multidimensional statistical analysis. (a and b) The figure shows a scatter plot where t [15] and t [19] represent the first and second principal components, respectively, and the ellipse represents the 95% confidence interval. Dots of the same color indicate individual biological replicates within a group, and the distribution of the dots reflects the variation between and within groups. (c and d): The figure shows the distribution of individual biological replicates within each group based on their variation in principal component 1 (t [15]) and principal component 2 (t [19]). Similar colors of dots represent individual biological replicates within the same group. (eand f) The figure shows the relationship between replacement retention (horizontal axis) and *R*^2^ and *Q*^2^ values (vertical axis). Green dots represent *R*^2^ values, blue dots represent *Q*^2^ values, and dashed lines represent regression lines for *R*^2^ and *Q*^2^. The upper right corner indicates the *R*^2^ and *Q*^2^ values of the original model where the replacement retention is equal to 1.

Significant metabolites between the groups were identified through a combination of univariate and multidimensional statistical analyses. The univariate analysis assessed differences in all detected metabolites in negative and positive ion modes, including unidentified metabolites. Differential metabolites with a fold change (FC) greater than 1.5 or less than 0.05 were illustrated in volcano plots (Figures 7(a) and 7(b)). The classification attribution of these differential metabolites was visualized using different colors (Figures 7(c) and 7(d)). Multidimensional statistical analysis was utilized to reduce the dimensionality of the collected data while retaining the maximum original information. Principal component analysis (PCA) was utilized to visualize the general distribution pattern of the samples among different groups and to assess the degree of variation among the samples within and between groups. The QC samples exhibited tighter clustering in both negative and positive ion modes, as depicted in Figures 8(a) and 8(b), indicating greater reproducibility across the experiments. In addition, orthogonal partial least-squares discriminant analysis (OPLS-DA) was conducted as a noise filtering method to improve the accuracy of the model. The scores from the OPLS-DA model in both negative and positive ion modes demonstrated a distinct differentiation between the experimental and control groups, with significant differences observed (Figures 8(c) and 8(d)). Furthermore, the model was subjected to a replacement test to ensure its validity and avoid overfitting. The results (Figures 8(e) and 8(f)) indicated a gradual decrease in both *R*^2^ and *Q*^2^ values of the stochastic model as the replacement retention decreased, affirming the reliability and robustness of the model.

### 3.9. Bioinformatics Analysis of Differential Metabolites

**Figure 9:**
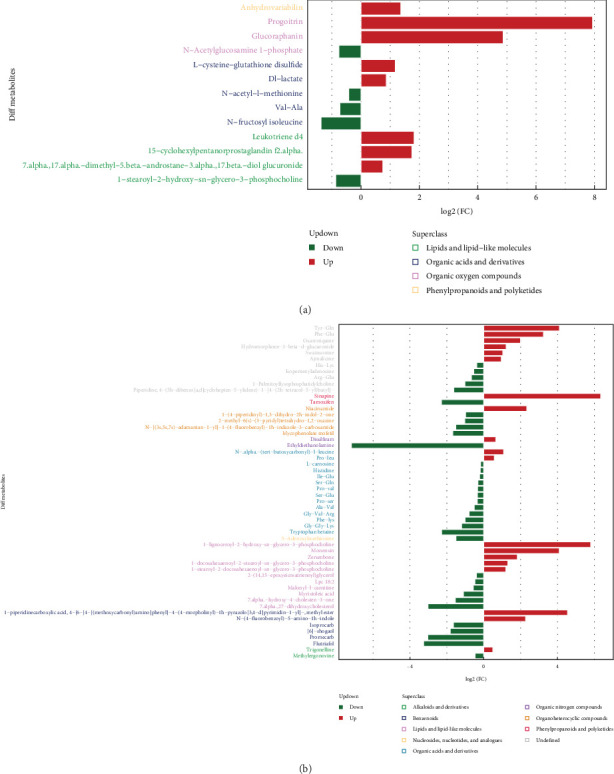
Significantly different metabolite expression differential ploidy analysis. (a and b) Negative/positive ion pattern significant differential metabolite expression differential multiplicity analysis. The horizontal coordinate of the graph indicates the log_2_FC value of the differential metabolite, which is the logarithmic value of the differential multiplier of the differential metabolite with a base of 2. The *Y*-axis of the plot represents significant differential metabolites. Upregulated metabolites are shown in red, and downregulated metabolites are shown in green.

To pinpoint metabolites that substantially contributed to model interpretation, Variable Importance for the Projection (VIP) values were extracted from the OPLS-DA model. Metabolites with VIP scores exceeding 1 were regarded as making significant contributions to model interpretation. The metabolites showing significant differences were visually represented with bar graphs, as shown in Figure 9. Progoitrin, Glucoraphanin, and Leukotriene d4 showed a marked increase in the negative ion mode, while Sinapine, 1-piperidinecarboxylic acid, 1-lignoceroyl-2-hydroxy-sn-glycero-3-phosphocholine, 4-(6-(4-((methoxycarbonyl)amino] phenyl)-4- (4-morpholinyl)-1h-pyrazolo(3,4-d)pyrimidin-1-yl)-, and methyl ester were significantly upregulated in the positive ion mode. These markedly different metabolites were further analyzed using cluster analysis, correlation analysis, and pathway analysis.

#### 3.9.1. Cluster Analysis

**Figure 10:**
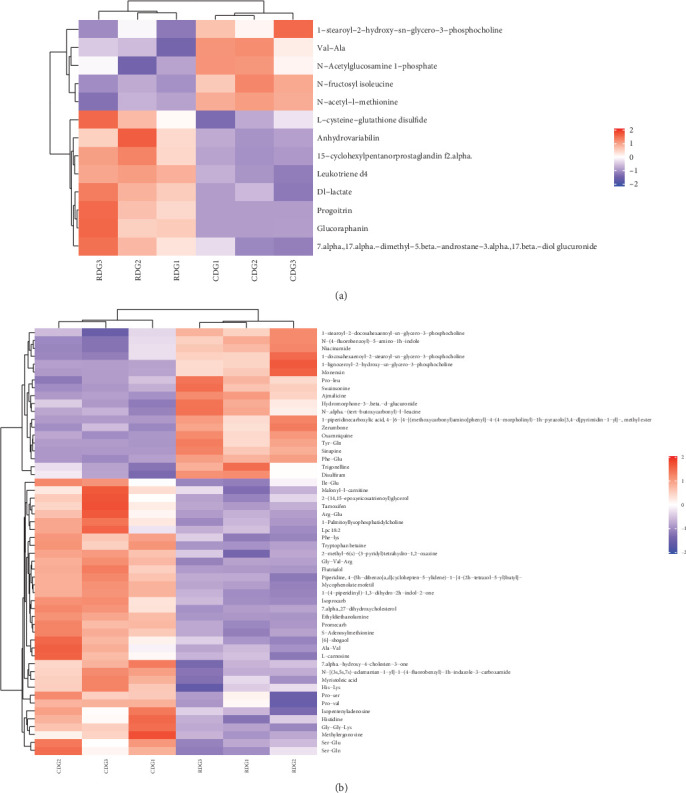
Heat map of hierarchical clustering for significantly different metabolites. (a and b) Negative/positive ion pattern significant difference metabolite hierarchical clustering heat map. The figure is a color-coded representation of differentially expressed metabolites (vertical axis) across a set of samples (horizontal axis). Red indicates upregulation, blue indicates downregulation and color intensity indicates the level of up or downregulation. Metabolites with similar expression patterns are clustered together on the left side of the figure.

To deepen our comprehension of the associations among samples and the diversity in metabolite expression patterns across samples, we conducted a hierarchical cluster analysis utilizing distance matrix calculations. This approach facilitated the identification of clusters of metabolites with similar expression patterns, implying potential shared functions or involvement in common metabolic processes or cellular pathways. The analysis focused on metabolites showing statistical significance, characterized by VIP values > 1 and *P*-values < 0.05. The outcomes of the hierarchical cluster analysis are illustrated in Figure 10.

#### 3.9.2. Correlation Analysis

**Figure 11:**
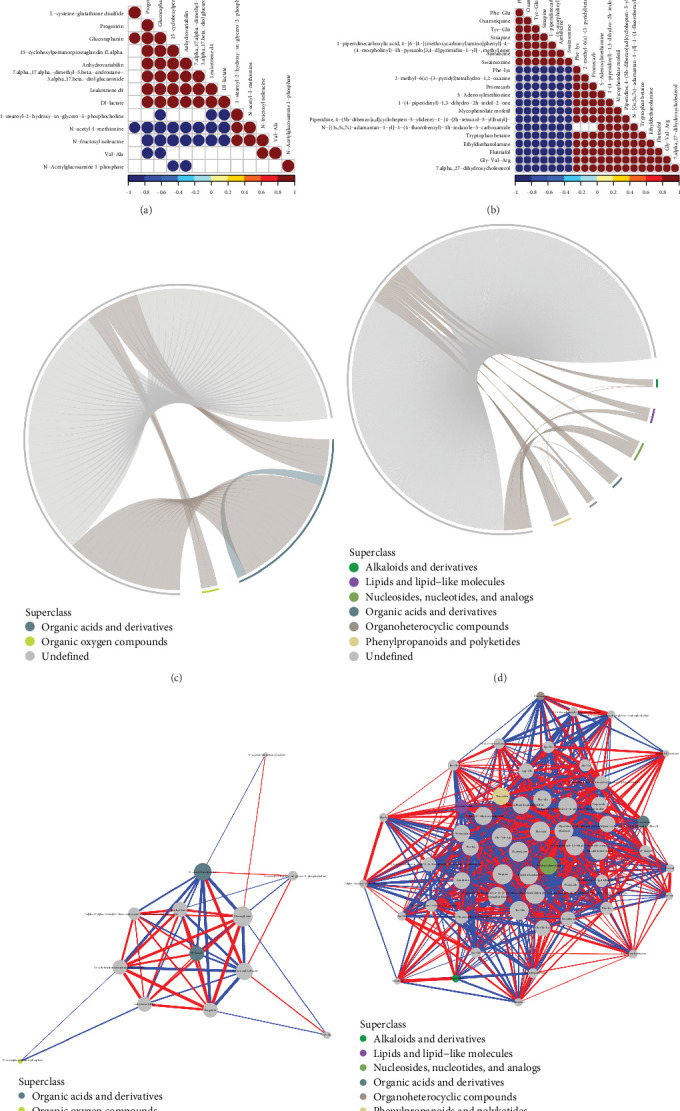
Correlation analysis. (a and b) The graph shows correlation coefficients between variables, where red indicates a positive correlation, blue indicates a negative correlation, and white indicates no correlation. The color shade indicates the strength of the correlation, whereas darker colors represent higher absolute values. The size of the dot indicates the significance of the correlation, where smaller *P*-values correspond to larger dots. (c and d) Each significantly different metabolite is represented by a starting point on the inner circle of the figure, and the outer circle shows the classification of these metabolites. colored arcs connecting the starting points indicate the correlation within each category of metabolites. The colors of the arcs match those of the corresponding subcategories. Gray arcs represent the correlations between metabolites of different categories. (e and f) The graph displays significantly different metabolites as dots, with their size indicating the degree of connectedness. The color of the line represents the correlation, with red indicating a positive correlation and blue indicating a negative correlation. The thickness of the line represents the strength of the correlation, with thicker lines representing larger correlation coefficients.

Correlation analysis serves as a valuable tool to explore the metabolic relationships among markedly different metabolites (VIP > 1, *P*value < 0.05) and provides additional insights into how metabolites are interregulated during biological changes. To present the results of the correlation analysis more visually, we created correlation heat maps, as depicted in Figures 11(a) and 11(b). To enhance the understanding of coregulatory associations among different metabolites, the connection matrices were transformed into chord diagrams and network diagrams, as illustrated in Figure 11(c)–11(f).

#### 3.9.3. KEGG Pathway Analysis

**Figure 12:**
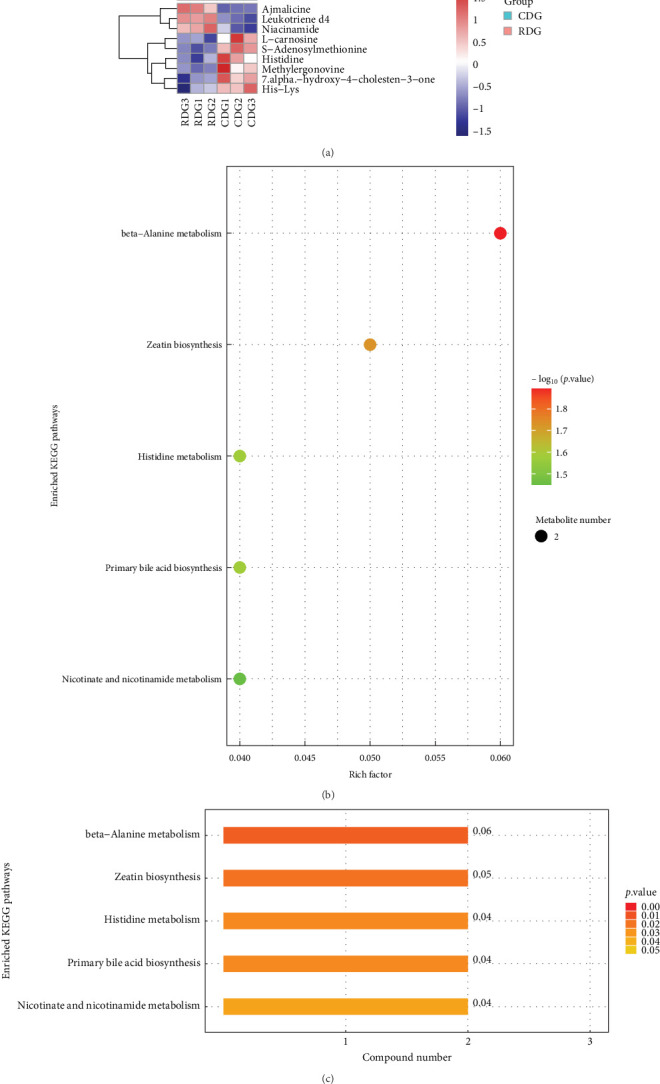
KEGG pathway analysis. (a) Heat map of differential metabolites for KEGG pathway clustering. Each row represents a differential metabolite with significant differential expression, while each column represents a sample set. The color scheme is as follows: red indicates upregulation, blue indicates downregulation and color shades indicate the degree of up and downregulation. The clustering of metabolites with similar expression patterns is represented on the left of the heatmap. (b) The bubble map shows KEGG pathways with significant enrichment. Each bubble represents a metabolic pathway, with size and horizontal position indicating the pathway's influence factor in topological analysis. The vertical position and color of the bubble represent the negative common logarithm of the *P*-value (i.e., −log_10_*P*-value), with darker colors indicating smaller *P*-values and more significant enrichment. (c) The bar graph shows the number of differentially expressed metabolites in each KEGG metabolic pathway, with each pathway represented on the vertical axis. The colour of the bars reflects the *P*-value of the enrichment analysis, with darker colours indicating more significant enrichment.

**Figure 13:**
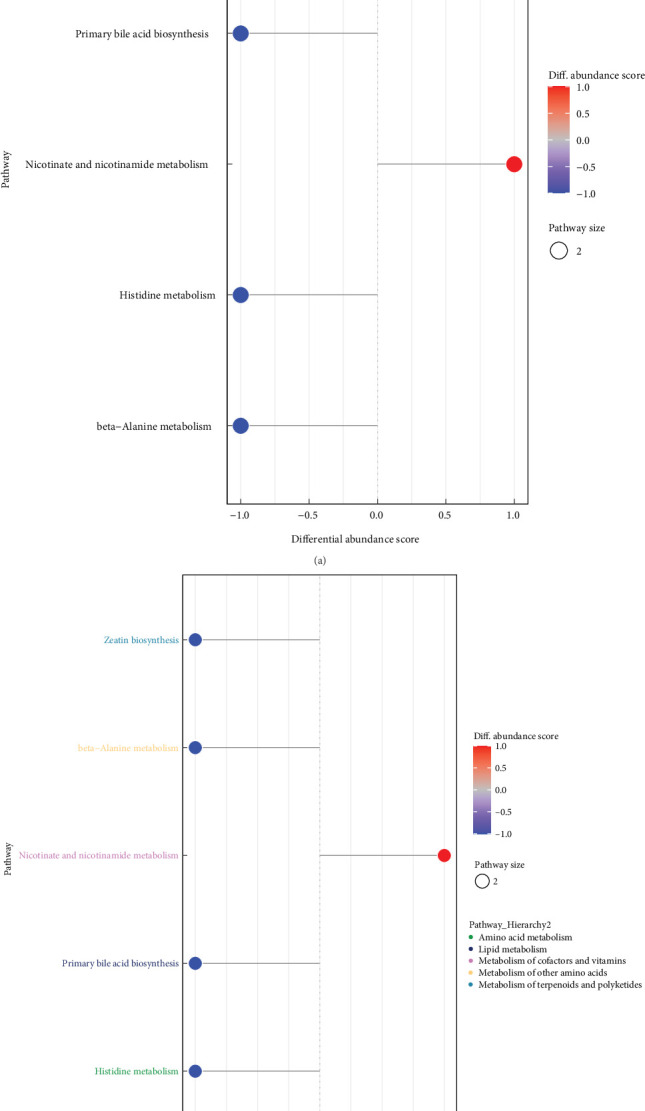
Differential abundance score analysis. (a and b) The plot shows the differential abundance scores of enriched metabolic pathways, categorized by pathway_Hierarchy. Each pathway is represented on the *Y*-axis, while the *X*-axis represents the differential abundance score (DA score), which reflects the total variation of all metabolites in the pathway. A score of 1 means all identified metabolites in the pathway are upregulated, while −1 means all metabolites are downregulated. The length of the line segment represents the absolute value of the DA score, and the dot size indicates the number of metabolites in the pathway. Darker red shades indicate upregulation of the pathway, while darker blue shades indicate downregulation.

The identified differential metabolites in the study underwent analysis using the KEGG database to explore the associated metabolic pathways. Heat maps were generated for KEGG metabolic pathways containing more than five differential metabolites, as illustrated in Figure 12 (a). Significant upregulation of Ajmalicine, Leukotriene d4, and Niacinamide metabolites were observed in the RDG group, while L-carnosine, Methylergonovine, and Histidine metabolites were markedly upregulated in the CDG group. Furthermore, metabolic pathways involving closely related species were used as a background for Fisher's Exact Test analysis to determine the significance level of metabolite enrichment for each pathway. The lower the *P*-value, the greater the significance of the variability of the metabolic pathway. The findings from the analysis (Figures 12(b) and 12(c)) revealed that the metabolic pathways were primarily enriched in beta-Alanine metabolism, Zeatin biosynthesis, Primary bile acid biosynthesis, Histidine metabolism, and Nicotinate and nicotinamide metabolism. Finally, the differential abundance scores were used to understand the trend of increasing/decreasing metabolites in a given pathway relative to the control samples. As shown in Figure 13 (a), one is increasing (>0.5 difference in abundance score, red) and four are decreasing (<−0.5, blue). The pathways that were elevated in the shrimp hepatopancreas involved the metabolism of nicotinic acid and nicotinamide, while most of the pathways that were decreased involved amino acid metabolism (Figure 13(a)). All the differential metabolic pathways were categorized by their upper-level Pathway_Hierarchy and then replotted as shown in Figure 13(b). The overall expression of metabolism of cofactors and vitamins pathways tended to be upregulated, while the metabolism of terpenoids and lipid metabolism, polyketides, metabolism of other amino acids, and amino acid metabolism pathways tended to be downregulated, as detailed in Table 4.

## 4. Discussion

Given its pivotal roles in both the digestive and immune systems of shrimp, the hepatopancreas serves as a dependable marker of crustacean well-being. It provides valuable information about the general well-being of the host [27, 28]. However, the hepatopancreas is susceptible to damage caused by pathogenic bacteria. For example, *V. harveyi* infection in *P. vanname* leads to abnormal arrangement of its hepatopancreas tubules and vacuolation of hepatocytes [29]. Infection with *V. parahaemolyticus* can cause hepatopancreas cell necrosis, abscission, and tissue degeneration in shrimp [30], ultimately resulting in AHPND and inhibiting the growth and health of shrimp. Historically, antibiotics have been commonly used to manage AHPND. However, emerging evidence suggests that probiotics can also be an effective approach to prevent and treat this disease [31, 32]. Nevertheless, the impact of probiotic Ep-M17 on shrimp hepatopancreas morphology remains unclear. Therefore, this study aimed to evaluate hepatopancreatic histology after both feeding and challenge trials to explore the impact of probiotic Ep-M17 on shrimp hepatopancreas. Contrasted with the control group, Ep-M17-fed shrimp exhibited a more intact hepatopancreatic basement membrane and a regular lumen structure of stellate ducts. Even after *V. parahaemolyticus* E1 infection, Ep-M17-fed shrimp maintained an intact hepatopancreatic basement membrane and well-preserved hepatic ducts, showing no signs of cell necrosis or severe cell damage. Therefore, feeding Ep-M17 could improve the morphology of the hepatopancreatic tissues and effectively reduce the damage caused by pathogenic bacteria to shrimp hepatopancreatic cells.

Increasing evidence demonstrates the advantageous impacts of probiotics on the growth performance, digestive enzyme activity, and disease resistance in aquatic animals. For instance, research has shown that dietary supplementation of *Bacillus* sp. enhanced feed conversion and growth performance in *Barbonymus gonionotus* [33]. An addition of *B. coagulans* and *L. lactis* was found could improve *Procambarus clarkii*'s growth performance, modulate its gut microbiota and enhance disease resistance [34]. *Clostridium butyricum* contributed to the increase of digestive enzyme activity of *P. vanname*, the enhancement of antioxidant capacity and the improvement of intestinal flora [35]. In this investigation, we explored the impact of dietary Ep-M17 supplementation on the nutritional immune-related enzyme in the hepatopancreas of *P. vannamei*. Findings revealed that Ep-M17 supplementation increased the activities of SOD, AKP, CAT, AMS, TRY, and LIP. Antioxidant enzymes such as CAT and SOD have a pivotal role in preventing oxidative stress and maintaining homeostasis overall homeostasis, effectively alleviating oxidative damage, deoxyribonucleic acid (DNA) and cellular damage, and even tissue damage and impaired cellular function caused by excessive production of reactive oxygen species (ROS) [36]. AKP, on the other hand, is a nonspecific phosphate hydrolase that regulates the immune system and contributes to defense by inducing stress [37]. Therefore, Ep-M17 shows potential in effectively preventing oxidative stress in the hepatopancreas and modulating the body's immune system.

The addition of probiotics to shrimp feed has been found to influence gene transcription in the intestine [38] and immune and metabolic genes in the hepatopancreas tissue [39], thus activating the immune response in the hepatopancreas and intestinal tract of shrimp, ultimately improving their disease resistance. The transcriptome analysis performed in this study identified a noteworthy enrichment of differentially expressed genes associated with immune response, heat shock protein, MAPK signaling pathway, and oxidoreductase activity in response to Ep-M17. Heat shock proteins (Hsps) are commonly recognized as danger signal biomarkers that stimulate the immune system to respond to unfavourable conditions. In addition, Hsps influence both pro- and anti-inflammatory responses and exerts unique effects on immune cells [40]. Oxidative changes are known to regulate various aspects of the immune response, especially the inflammatory pathway [41, 42]. For instance, Alkylglycerol monooxygenase (AGMO) can regulate the production of macrophage platelet-activating factor (PAF) by modulating cytolytic-PAF levels [43]. The enriched expression of these genes demonstrated that Ep-M17 can significantly upregulate the immune genes in *P. vanname* hepatopancreas, facilitating an anti-inflammatory response in cells and effectively regulating the immune response, thereby improving the shrimp's immune capacity. Moreover, KEGG annotation and enrichment analysis revealed that Ep-M17 primarily affects antigen processing and presentation, MAPK signaling pathway, PI3K-Akt signaling pathway, and necrotic apoptotic cell death pathways. Antigen processing and presentation are critical for adaptive immunity [44] and the activation of T cells [45], which play essential roles in defending against pathogens [46]. The MAPK pathway is also important for host defense against pathogens [47]. Specifically, p38 MAPK signaling is required for the transcriptional activation of NF-*κ*B [48], which triggers the production of inflammatory cytokines and initiates innate immune responses [49]. The enrichment of differential genes in these pathways suggests that Ep-M17 can regulate adaptive immunity in shrimp hepatopancreas through the antigen processing and presentation pathways. Furthermore, it can influence MAPK signaling pathways associated with the initiation of innate immunity and the secretion of inflammatory cytokines, thereby affecting the host's immune response.

Probiotics have been shown to modulate the host's immune system, reduce inflammation, and improve various aspects of the body's metabolism, growth, fatty acid profile, and oxidative stability [50]. In this study, the metabolomic analysis revealed that shrimp fed Ep-M17 had significantly higher levels of associated hepatopancreas metabolites involved in the metabolic pathways of beta-alanine metabolism, Zeatin biosynthesis, Histidine metabolism, Nicotinate and nicotinamide metabolism, and Primary bile acid biosynthesis. *β*-alanine is known to activate the NF-*κ*B signaling pathway, which contributes to the inflammatory response, and it specifically activates G protein-coupled receptors (MrgprD) in the organism, thereby regulating inflammation triggered by bacterial infections [51]. Histidine decarboxylase is responsible for the production of histamine, which can activate various histamine receptors on target cells and modulate a range of physiological and immunological processes. Histamine has been observed to induce platelet aggregation and modulate Th2 cell activity by reducing IL-12 levels while increasing IL-10 production [52]. The upregulation of these metabolic pathways suggests that Ep-M17 can facilitate the production of *β*-alanine, activate the NF-*κ*B signaling pathway, and thereby regulate inflammation. In addition, Ep-M17 may influence other key immune metabolites, ultimately enhancing the body's anti-inflammatory and antimicrobial capacity, which in turn promotes shrimp growth.

## 5. Conclusion

The principal purpose of this study was to explore the protective mechanism of *L. plantarum* Ep-M17 on the hepatopancreas of *P. vannamei*. The results demonstrated that supplementing *P. vannamei*'s diet with *L. plantarum* Ep-M17 effectively improves the stability of the hepatopancreatic tissue. Furthermore, Ep-M17 elevates the activity of metabolism and immune-related enzymes in the hepatopancreas, while also significantly upregulating genes associated with immune response, antigen processing and presentation, and the MAPK signaling pathway. In addition, Ep-M17 promotes the activity of metabolites in the *P. vannamei* hepatopancreas that are known for their anti-inflammatory and cellular damage-preventive effects, particularly within the pathways of *β*-alanine metabolism and histidine metabolism. These findings highlight the promising potential of Ep-M17 as a probiotic agent for the prevention and management of aquatic diseases.

## Figures and Tables

**Table 1:** Statics of sequencing.

Sample	Reads no.	Bases (bp)	Q30 (bp)	*N* (%)	Q20 (%)	Q30 (%)	Clean reads no.	Clean data (bp)	Clean reads (%)	Clean data (%)
CDG-1	44,944,802	6,786,665,102	6,255,074,092	0.000695	96.91	92.16	42,548,068	6,424,758,268	94.66	94.66
CDG-2	46,489,684	7,019,942,284	6,496,633,473	0.000712	97.05	92.54	44,066,664	6,654,066,264	94.78	94.78
CDG-3	39,163,888	5,913,747,088	5,413,467,606	0.000721	96.62	91.54	37,073,016	5,598,025,416	94.66	94.66
RDG-1	39,916,996	6,027,466,396	5,577,144,286	0.000712	97.11	92.52	37,789,824	5,706,263,424	94.67	94.67
RDG-2	45,287,730	6,838,447,230	6,274,854,056	0.000723	96.72	91.75	42,842,026	6,469,145,926	94.59	94.59
RDG-3	40,577,070	6,127,137,570	5,610,999,013	0.000722	96.68	91.57	38,444,440	5,805,110,440	94.74	94.74

Sample: Sample name; No.: Total number of Reads; Bases (bp): Total number of Bases; Q30 (bp): Total number of bases for which recognition accuracy is above 99.9%; N (%): Percentage of fuzzy bases; Q20 (%): The percentage of bases for which recognition accuracy is above 99%; Q30 (%): Percentage of bases for which recognition accuracy is above 99.9%; Clean Reads No: High-quality sequence read number; Clean Data (bp): High-quality sequence base number; Clean Reads %: High-quality Reads take up the percentage of sequencing Reads; Clean Data %: The percentage of high-quality sequence bases in sequencing bases.

**Table 2:** GO enrichment of immune-related genes.

ID	Fold change	*P* value	Homologues function	Species
Immune response				
TRINITY_DN13590_c0_g1	19.27	0.02729	C-type lectin 1	*Penaeus vannamei*
TRINITY_DN497_c2_g1	2.24	0.00137	Dual 3&apos;,5&apos;-cyclic-AMP and—GMP phosphodiesterase 11	*P. vannamei*
TRINITY_DN6466_c1_g1	2.08	0.00054	Caspase-8-like	*Penaeus monodon*
TRINITY_DN10125_c0_g1	Inf	0.00222	Hypothetical protein EGW08_020278	*Elysia chlorotica*
TRINITY_DN7113_c0_g1	63.04	0.00419	Hypothetical protein L596_006287	*Steinernema carpocapsae*
TRINITY_DN1907_c0_g1	Inf	0.00525	Hypothetical protein CAPTEDRAFT_183889	*Capitella teleta*
TRINITY_DN1625_c4_g1	76.72	0.01233	Sequestosome-1	*Toxocara canis*
TRINITY_DN18993_c3_g1	Inf	0.00343	R27a protein	*Phallusia mammillata*
TRINITY_DN76030_c0_g1	Inf	0.03414	Predicted protein	*Nematostella vectensis*
TRINITY_DN5401_c0_g1	Inf	0.04592	Heat Shock 70kda Protein 5-like	*Varroa destructor*
TRINITY_DN2659_c0_g1	3.08	0.00015	Tyrosine-protein phosphatase Lar-like	*P. monodon*
TRINITY_DN1605_c0_g1	2.48	0.00003	Baculoviral IAP repeat-containing protein 8-like	*Penaeus vannamei*
Heat shock protein				
TRINITY_DN10612_c0_g3	Inf	0.00002	Inducible heat shock protein 70	*Tigriopus japonicus*
TRINITY_DN10612_c0_g2	Inf	0.00007	Inducible heat shock protein 70	*T. japonicus*
TRINITY_DN1801_c0_g1	173.92	0.00030	Heat shock protein 90	*Plakobranchus ocellatus*
TRINITY_DN31166_c0_g1	Inf	0.00130	Heat shock cognate 70 kda protein 2-like	*Artibeus jamaicensis*
MAPK signaling pathway				
TRINITY_DN3105_c0_g1	Inf	0.00649	Serine protease 2	*Costelytra zealandica*
TRINITY_DN5232_c0_g1	2.59	0.00137	LOW QUALITY PROTEIN: probable phosphoserine aminotransferase	*P. vannamei*
TRINITY_DN16109_c0_g1	2.44	0.04529	N-terminal Xaa-Pro-Lys N-methyltransferase 1-like	*P. monodon*
Oxidoreductase activity				
TRINITY_DN85_c1_g1	2.65	0.00000	Putative alkylglycerol monooxygenase-like	*P. vannamei*
TRINITY_DN4904_c2_g1	2.04	0.00052	Alkylglycerol monooxygenase-like isoform X1	*P. monodon*
TRINITY_DN5015_c0_g1	2.51	0.01219	Gamma-butyrobetaine dioxygenase-like	*P. vannamei*
TRINITY_DN3014_c0_g1	2.33	0.01705	LOW QUALITY PROTEIN: phenoloxidase 3-like	*P. vannamei*
TRINITY_DN3935_c0_g1	Inf	0.02168	Unnamed protein product	*Didymodactylos carnosus*
TRINITY_DN4486_c0_g1	Inf	0.02479	Glucosamine-6-phosphate isomerase 1 isoform X1	*Choloepus didactylus*
TRINITY_DN9040_c0_g1	2.10	0.03642	Protein disulfide-isomerase A4-like	*P. vannamei*
TRINITY_DN6586_c0_g1	19.81	0.03642	Protein disulfide-isomerase	*Elysia marginata*
TRINITY_DN79418_c0_g1	Inf	0.03772	Aldo-keto reductase family 1 member A1-B	*Polypterus senegalus*
TRINITY_DN6846_c0_g2	2.17	0.02998	Hypothetical protein C7M84_010294	*P. vannamei*
Transmembrane signaling receptor activity				
TRINITY_DN10469_c0_g1	3.21	0.03763	Uncharacterized protein LOC113805974	*P. vannamei*
TRINITY_DN13590_c0_g1	19.27	0.02729	C-type lectin 1	*P. vannamei*
TRINITY_DN14206_c0_g1	5.81	0.02378	Octopamine receptor beta-2R-like	*P. vannamei*
TRINITY_DN1990_c0_g1	2.57	0.03178	Insulin receptor-related protein-like	*P. monodon*
TRINITY_DN2659_c0_g1	3.08	0.00015	Tyrosine-protein phosphatase Lar-like	*P. monodon*
TRINITY_DN33806_c0_g1	11.30	0.04639	Hypothetical protein C7M84_001710	*P. vannamei*
TRINITY_DN7471_c0_g1	4.83	0.03848	G-protein coupled receptor moody-like	*P. vannamei*
Autophagy				
TRINITY_DN10125_c0_g1	Inf	0.00222	Hypothetical protein EGW08_020278	*E. chlorotica*
TRINITY_DN1625_c4_g1	76.72	0.01233	Sequestosome-1	*T. canis*
TRINITY_DN1907_c0_g1	Inf	0.00525	Hypothetical protein CAPTEDRAFT_183889	*C. teleta*
TRINITY_DN1990_c0_g1	2.57	0.03178	Insulin receptor-related protein-like	*P. monodon*
TRINITY_DN23565_c0_g1	22.65	0.01439	Elongation factor 1-alpha 1	*Microcaecilia unicolor*
TRINITY_DN31166_c0_g1	Inf	0.00130	Heat shock cognate 70 kda protein 2-like	*A. jamaicensis*
TRINITY_DN6466_c1_g1	2.08	0.00054	Caspase-8-like	*P. monodon*
TRINITY_DN7035_c0_g1	3.74	0.00000	LOW QUALITY PROTEIN: elongation factor 1-alpha-like	*P. monodon*
TRINITY_DN7113_c0_g1	63.04	0.00419	Hypothetical protein L596_006287	*S. carpocapsae*
TRINITY_DN8760_c0_g1	12.53	0.01795	Recname: Full = Elongation factor 1-alpha 1; Short = EF-1-alpha-1	*Oscheius tipulae*

**Table 3:** KEGG enrichment of immune-related pathways.

ID	Fold change	*P* value	Homologues function	Species
Antigen processing and presentation				
TRINITY_DN1801_c0_g1	173.92	0.00030	Heat shock protein 90	*P. ocellatus*
TRINITY_DN5401_c0_g1	Inf	0.04592	78 kda glucose-regulated protein-like	*Varroa jacobsoni*
TRINITY_DN7113_c0_g1	63.04	0.00419	Hypothetical protein L596_006287	*S. carpocapsae*
TRINITY_DN10612_c0_g2	Inf	0.00007	Inducible heat shock protein 70	*T. japonicus*
TRINITY_DN10612_c0_g3	Inf	0.00002	Inducible heat shock protein 70	*T. japonicus*
TRINITY_DN31166_c0_g1	Inf	0.00130	Heat shock cognate 70 kda protein 2-like	*A. jamaicensis*
MAPK signaling pathway				
TRINITY_DN1990_c0_g1	2.57	0.03178	Insulin receptor-related protein-like	*P. monodon*
TRINITY_DN7113_c0_g1	63.04	0.00419	Hypothetical protein L596_006287	*S. carpocapsae*
TRINITY_DN10612_c0_g2	Inf	0.00007	Inducible heat shock protein 70	*T. japonicus*
TRINITY_DN10612_c0_g3	Inf	0.00002	Inducible heat shock protein 70	*T. japonicus*
TRINITY_DN31166_c0_g1	Inf	0.00130	Heat shock cognate 70 kda protein 2-like	*A. jamaicensis*
PI3K-Akt signaling pathway				
TRINITY_DN1990_c0_g1	1.61	0.01739	Uncharacterized protein LOC113801216	*P. vannamei*
TRINITY_DN2659_c0_g1	3.08	0.00015	Tyrosine-protein phosphatase Lar-like	*P. monodon*
TRINITY_DN1801_c0_g1	173.92	0.00030	Heat shock protein 90	*P. ocellatus*
Necroptosis				
TRINITY_DN1801_c0_g1	173.92	0.00030	Heat shock protein 90	*P. ocellatus*
TRINITY_DN1907_c0_g1	Inf	0.00525	Hypothetical protein CAPTEDRAFT_183889	*C. teleta*
TRINITY_DN3799_c0_g1	Inf	0.00076	PREDICTED: histone H2A-like	*Saccoglossus kowalevskii*
TRINITY_DN6174_c0_g1	8.19	0.04836	Glycogen phosphorylase, brain form isoform X1	*Chelonia mydas*
Apoptosis-multiple species				
TRINITY_DN1605_c0_g1	2.48	0.00003	Putative baculoviral IAP repeat-containing protein 7 isoform X2	*P. vannamei*
TRINITY_DN6466_c1_g1	2.08	0.00054	Caspase-8-like	*P. monodon*
Neutrophil extracellular trap formation				
TRINITY_DN3799_c0_g1	Inf	0.00076	PREDICTED: histone H2A-like	*S. kowalevskii*
TRINITY_DN3863_c1_g1	11.65	0.04836	Actin, clone 403	*Araneus ventricosus*
TRINITY_DN9546_c0_g1	Inf	0.01737	Putative histone H2B-like, partial	*Cotesia chilonis*
TRINITY_DN3778_c0_g1	Inf	0.00384	PREDICTED: uncharacterized protein LOC105526835	*Colobus angolensis palliatus*

**Table 4:** KEGG metabolic pathway analysis.

KEGG ID	Pathway	Name	Change
C16427	Zeatin biosynthesis	Isopentenyladenosine	Down
C16427	Biosynthesis of secondary metabolites	Isopentenyladenosine	Down
C10173	Tropane, piperidine, and pyridine alkaloid biosynthesis	Swainsonine	Up
C09024	Indole alkaloid biosynthesis	Ajmalicine	Up
C09024	Metabolic pathways	Ajmalicine	Up
C09024	Biosynthesis of secondary metabolites	Ajmalicine	Up
C07108	Drug metabolism—cytochrome P450	Tamoxifen	Down
C06341	Primary bile acid biosynthesis	7.alpha.,27-dihydroxycholesterol	Down
C05951	Arachidonic acid metabolism	Leukotriene d4	Up
C05951	Metabolic pathways	Leukotriene d4	Up
C05951	Neuroactive ligand-receptor interaction	Leukotriene d4	Up
C05951	Fc epsilon RI signaling pathway	Leukotriene d4	Up
C05455	Primary bile acid biosynthesis	7.alpha.-hydroxy-4-cholesten-3-one	Down
C05455	Metabolic pathways	7.alpha.-hydroxy-4-cholesten-3-one	Down
C04230	Glycerophospholipid metabolism	1-stearoyl-2-hydroxy-sn-glycero-3-phosphocholine	Down
C01004	Nicotinate and nicotinamide metabolism	Trigonelline	Up
C00933	Phenylpropanoid biosynthesis	Sinapine	Up
C00884	Arginine and proline metabolism	Methylergonovine	Down
C00884	Metabolic pathways	Methylergonovine	Down
C00387	Purine metabolism	His-Lys	Down
C00387	Metabolic pathways	His-Lys	Down
C00387	ABC transporters	His-Lys	Down
C00386	Histidine metabolism	L-carnosine	Down
C00386	beta-Alanine metabolism	L-carnosine	Down
C00386	Metabolic pathways	L-carnosine	Down
C00153	Nicotinate and nicotinamide metabolism	Niacinamide	Up
C00153	Metabolic pathways	Niacinamide	Up
C00153	Longevity regulating pathway—worm	Niacinamide	Up
C00153	Vitamin digestion and absorption	Niacinamide	Up
C00135	Histidine metabolism	Histidine	Down
C00135	Staurosporine biosynthesis	Histidine	Down
C00135	beta-Alanine metabolism	Histidine	Down
C00135	Aminoacyl-tRNA biosynthesis	Histidine	Down
C00135	Metabolic pathways	Histidine	Down
C00135	Biosynthesis of secondary metabolites	Histidine	Down
C00135	Biosynthesis of amino acids	Histidine	Down
C00135	ABC transporters	Histidine	Down
C00135	Protein digestion and absorption	Histidine	Down
C00019	Monobactam biosynthesis	S-Adenosylmethionine	Down
C00019	Cysteine and methionine metabolism	S-Adenosylmethionine	Down
C00019	Arginine and proline metabolism	S-Adenosylmethionine	Down
C00019	Zeatin biosynthesis	S-Adenosylmethionine	Down
C00019	Metabolic pathways	S-Adenosylmethionine	Down
C00019	Biosynthesis of secondary metabolites	S-Adenosylmethionine	Down
C00019	Biosynthesis of amino acids	S-Adenosylmethionine	Down
C00019	Sulfur relay system	S-Adenosylmethionine	Down

## Data Availability

Data will be made available on request.
